# Carotid Paraganglioma in Adolescence-Clinical Picture-Surgical Technique and Review of the Literature

**DOI:** 10.1155/2019/6182783

**Published:** 2019-03-10

**Authors:** Thomas Kotsis, Panagitsa Christoforou, Constantinos Nastos

**Affiliations:** ^1^Vascular Division, 2nd Department of Surgery, National & Kapodistrian University of Athens Medical School, “Aretaieion” Hospital, Athens, Greece; ^2^2nd Department of Surgery, National & Kapodistrian University of Athens Medical School, “Aretaieion” Hospital, Athens, Greece

## Abstract

Paraganglia are clusters of cells originating from the neural crest with histological and cytochemical characteristics of neuroendocrine cells. Paragangliomas of the head and neck represent less than 0.5% of all head and neck tumors and they usually occur between the ages of 40 and 50. Paragangliomas in childhood and in adolescence are extremely rare; only 23 case reports have been reported in the recent literature. In childhood, the estimation of malignant potential is 3–10%; therefore, early diagnosis and treatment of carotid body paragangliomas are mandatory. However, due to the rarity of these lesions in young patients, they are often not included in the differential diagnosis of solid masses in the neck area, a fact that may lead to misdiagnosis or delay in treatment. We present, herein, two extremely rare cases of patients in adolescence who were diagnosed with a carotid body paraganglioma and were treated surgically in our unit. One of the patients was diagnosed and treated at the age of 15 years while the other had a long-standing tumor in the neck that was followed up by a general surgery outpatient service as a branchial cleft cyst at the age of 15 years and was eventually treated surgically 8 years later. Carotid body tumor was not considered in the initial differential diagnosis because of its rarity at this age range.

## 1. Introduction

Paraganglia are clusters of cells with histological and cytochemical characteristics of neuroendocrine cells originating from the neural crest [1]. Paragangliomas of the head and neck represent less than 0.5% of all head and neck tumors [2]. Sporadic paragangliomas appear more frequent in female than male, at the age of 40 to 50 years, while familial paragangliomas present earlier and more commonly in male [3]. Pediatric paragangliomas are even more rare entities and only a few cases have been reported in the recent literature [4].

We present two cases of carotid body paragangliomas (CBP) that occurred during adolescence; one of the patients had a delayed treatment, as a neck mass initially observed at childhood was considered to be a branchial cleft cyst, for which he had annual surveillance at a general surgery outpatient service.

The clinical picture, the imaging investigations, the differential diagnosis, and the surgical treatment are discussed in the current manuscript, along with a review of similar published cases.

## 2. Material and Methods

### 2.1. Search Methods

We searched the PubMed and Cochrane Library databases with no language restrictions. Studies or case reports were published between 1968 and 2018.

### 2.2. Selection Criteria

We performed a literature search with the keywords “Paraganglioma”, “Carotid body paraganglioma”, “Head and neck paraganglioma”, and “Chemodectoma”, without limitations to the dates or the article types. According to the database and the used terms, many studies and incidents were reported. Our research focuses on carotid body paragangliomas in adolescence, so more specific investigation with the keywords “adolescence carotid body paraganglioma” and “pediatric carotid body paraganglioma” has revealed only 23 published case reports.

### 2.3. Data Collection and Analysis

Two reviewers independently assessed the risk of bias. They extracted the characteristics and results of the various studies that were discussed. In case of any disagreements that had occurred during data extraction, the third reviewer was asked to decide.

### 2.4. Main Results

During the literature search, many investigations found to be referred to multiple incidents, including a number of pediatric people treated with CBP. In some of these series, there is no reference of the exact number of pediatric patients being treated, nor the preoperative, intraoperative, and postoperative management of these patients.

The search produced 21 papers that were reviewed (Table 1). Information about the number of cases with neck paragangliomas at pediatric age group, the year of publication, the patients age and gender, the presenting symptoms, the size of the tumor, the Shamblin type, the malignancy, the cranial nerve injury, the treatment, and the follow-up period with no complications were identified and tabulated.

## 3. Case Reports

We report two cases of carotid paraganglioma in young age that have been operated in our department in the last 14 years, with a diagnosis of carotid body paragangliomas diagnosed and treated during adolescence, by the same surgeon (first author).

### 3.1. Case 1

**Figure 1:**
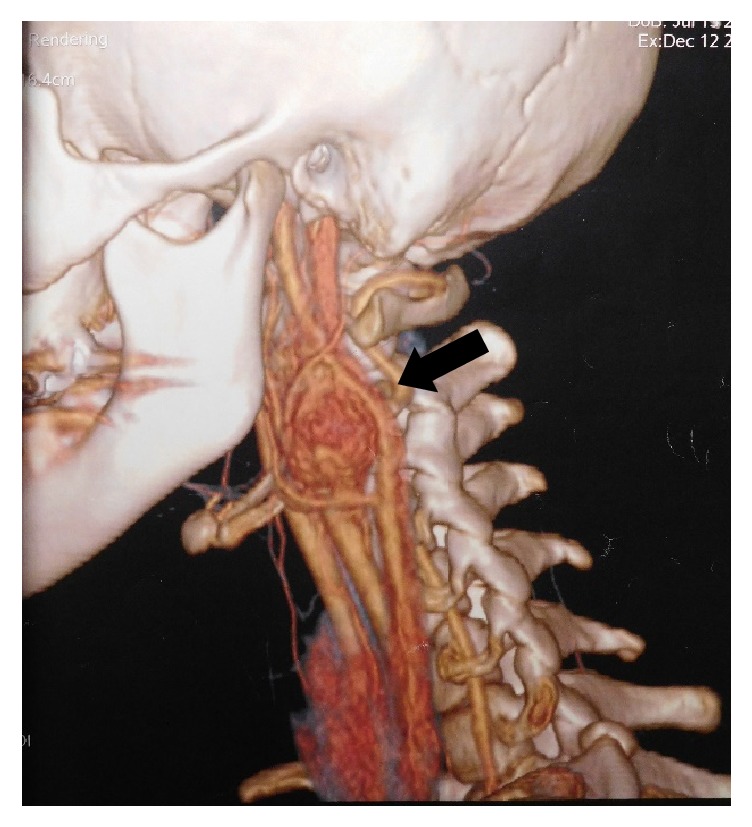
3-dimensional (3D) cervical-computed tomography angiography (CTA), lyre sign (arrow).

**Figure 2:**
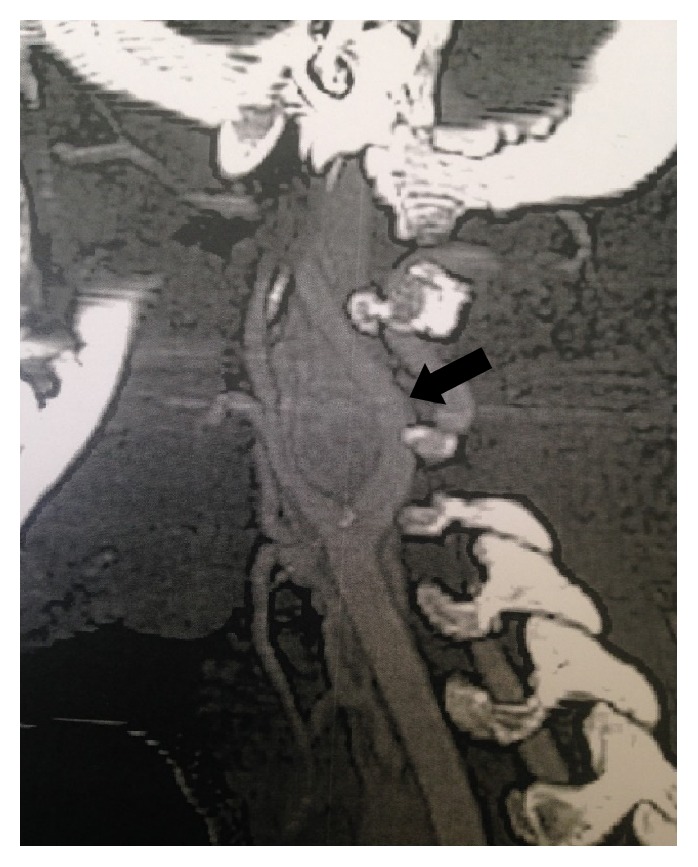
CTA-carotid body paraganglioma (CBP), lyre sign (arrow).

**Figure 3:**
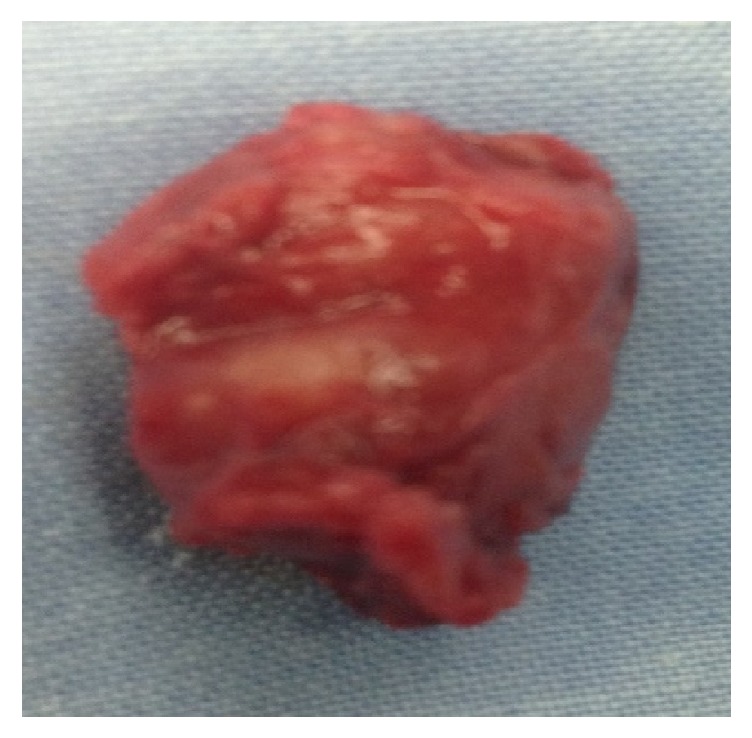
Paraganglioma of carotid tumor after surgical resection.

**Figure 4:**
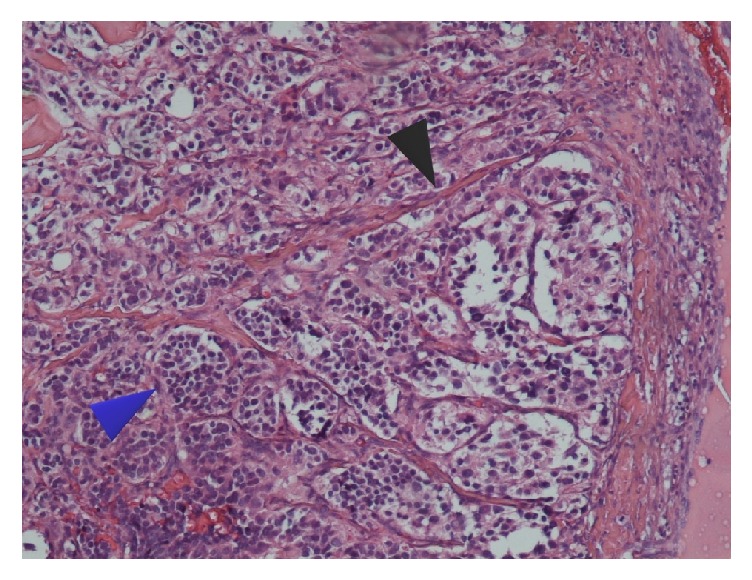
Histopathological appearance of CBP (16-year-old teenage girl) showing “zellballen” growth pattern of paragangliomas with central round/oval chief cells containing abundant eosinophilic granular or vacuolated cytoplasm, uniform nuclei with dispersed chromatin-nests of cells (blue arrow). Prominent fibrovascular stroma separates nests (black arrow).

A 16-year-old girl, with a free past medical history, presented with a palpable mass on the left side of the neck, complaining of dysphagia and cervical pain associated with dizziness. Neurologic examination was unremarkable and diagnostic tests for Epstein–Barr infection were negative. The patient underwent a thyroid ultrasound (US), which did not reveal any significant findings from the thyroid gland. However, a 27.5mm oval shaped, well-defined, hypoechoic, solid lesion was found at the left carotid triangle. The lesion showed high vascularity. Magnetic resonance imaging (MRI) of the neck followed showing an ovoid mass measuring 26x21x30mm between the left internal and external carotid arteries. A computed tomography angiography (CTA) was also performed with similar findings, suggesting the diagnosis of a CBP (Figures 1 and 2). The patient was electively admitted in our vascular unit in order to be treated surgically. A mass 2.5 cm was removed which was classified as Shamblin II (Figure 3). The histopathology results showed “zellballen” growth pattern of paraganglioma with central round/oval chief cells containing abundant eosinophilic granular or vacuolated cytoplasm, uniform nuclei with dispersed chromatin-nests of cells. Prominent fibrovascular stroma separated characteristic nests of paraganglioma tissue and there was no evidence of malignancy (Figure 4).

The patient had no postoperative neurologic symptoms except a transient episode of left parietal hypoesthesia. This was further investigated with an MRI of the brain and carotid ultrasound, which did not reveal sinister findings.

She had an uneventful postoperative recovery and was subsequently discharged on the 3rd postoperative day (POD).

### 3.2. Case 2

**Figure 5:**
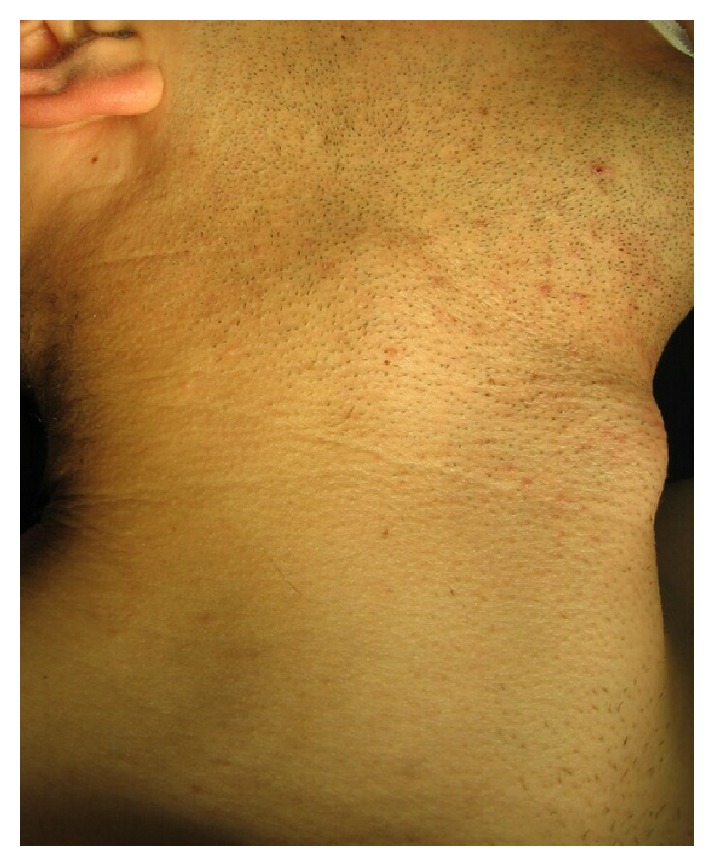
Painless palpable well-defined mass in the right carotid triangle.

**Figure 6:**
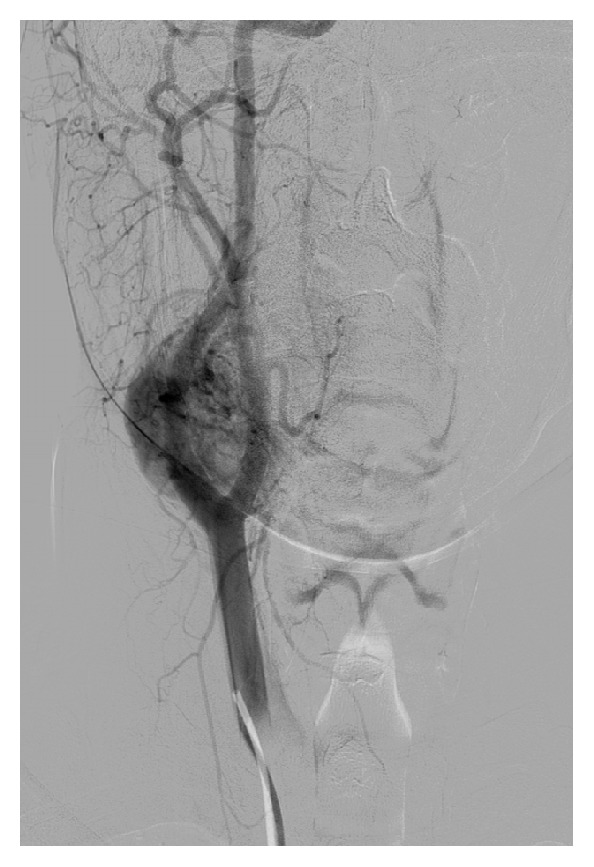
Digital subtraction angiography (DSA) revealed a carotid body paraganglioma in the right carotid bifurcation.

**Figure 7:**
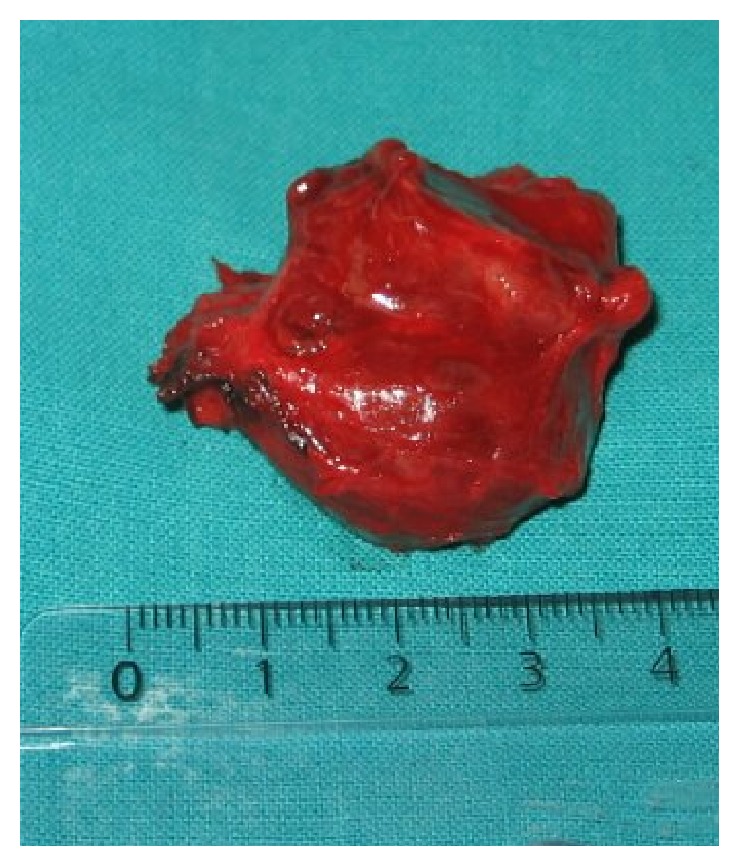
Paraganglioma of carotid tumor after surgical resection was 3 cm in diameter.

**Figure 8:**
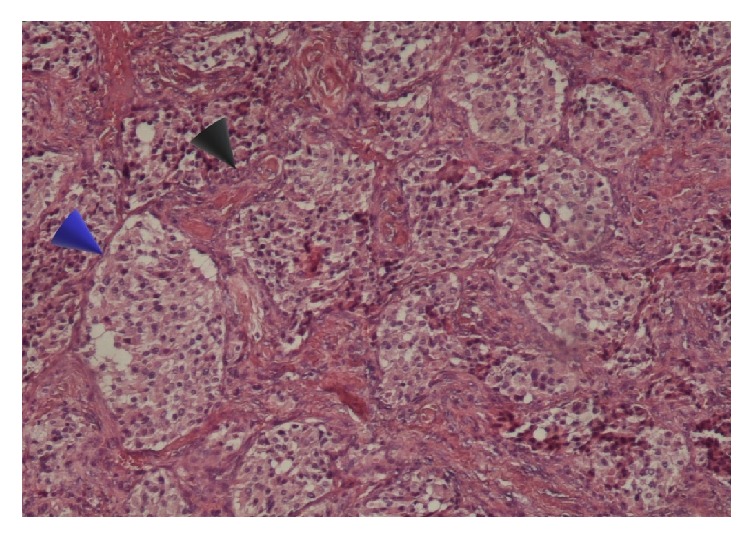
Histopathological appearance of CBP (15-year-old teenage boy) showing “zellballen” growth pattern of paragangliomas (blue arrow). Prominent fibrovascular stroma separates nests (black arrow).

A 15-year-old teenage boy initially presented in a district hospital, with a swelling at the right side of his neck, without any significant clinical symptoms. Although full details of the work-up performed at the time are not available, he was diagnosed with a branchial cleft cyst and was offered surveillance with follow-up imaging. Eight years later, he visited our unit. Physical examination revealed a painless palpable well-defined mass (Figure 5) within the right carotid triangle with positive Fontaine and Kocher I signs [1]. There was no palpable lymphadenopathy. An ultrasound scan was performed depicting a solid mass of mixed echogenicity in the right carotid triangle echogenicity in the left carotid triangle. This was suspected to be neurogenic in origin because of its location. A digital subtraction angiography (DSA) (Figure 6) followed, which revealed a 60x35mm protruding mass in the right carotid bifurcation, causing local compressive effects and posterior displacement of the vessels. The patient was admitted in our unit in order to be treated surgically. During the operation a large CBP was identified as seen in Figure 7. The tumor was classified as Shamblin II and was completely excised. Histopathology study showed a 3 cm carotid body paraganglioma with characteristic “zellballen” growth pattern and cell nests surrounded by prominent fibrovascular stroma, with no evidence of malignancy (Figure 8). The patient had an unremarkable recovery and was discharged home on the second POD.

**Figure 9:**
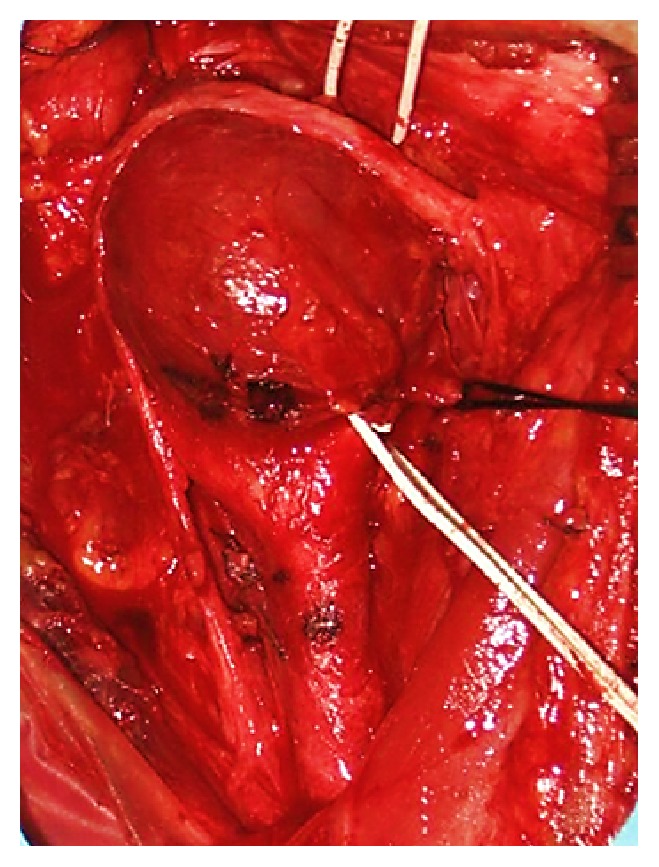
The right carotid triangle with carotid body paraganglioma.

**Figure 10:**
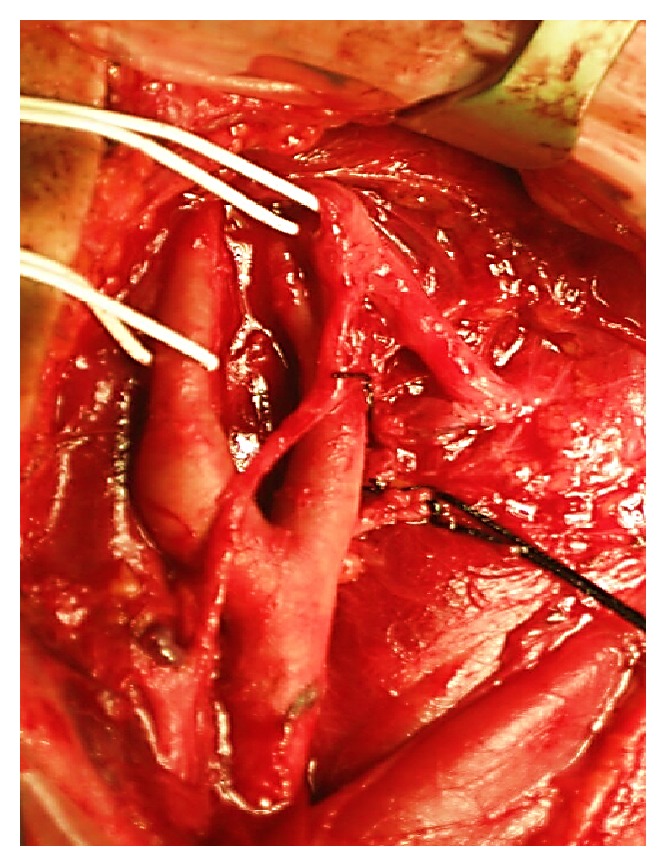
The carotid body paraganglioma of the right carotid triangle was completely excised, with preservation of the hypoglossal and vagus nerve.

The surgical technique adopted in both patients consisted of an oblique incision along the sternal head of the left sternocleidomastoid muscle under general anesthesia. Our strategy was to expose, dissect, and isolate the proximal common carotid artery using a vascular tape. The ansa cervicalis was also exposed early in the operation. The carotid bifurcation was exposed in a caudocranial approach. The proximal external and the proximal internal carotid arteries were isolated and controlled with vessel loops. Dissection was extended to the level of the digastor muscle in order to expose and control the distal internal carotid artery. During this process, the hypoglossal nerve was identified and preserved, by following the ansa cervicalis. The external carotid artery was cross-clamped temporarily. Finally, the tumor was removed with sharp dissection from the bifurcation with meticulous technique in order to avoid injury to the internal carotid artery and the cranial nerves. The reported plane of dissection reported as a white interface plane between the tumor and the vessels was not identified in the first patient as the tumor was severely adhering to the vessel wall and was typically found in the second patient. Following the removal of the CBP, the carotid sheath and platysma were approximated and the skin closed with a continuous subcuticular suture, after performing meticulous hemostasis (Figures 9 and 10).

Both patients had an annual postoperative follow-up with cervical ultrasound and carotid duplex ultrasonography with no evidence of local recurrence. Familial disease was excluded clinically, by screening the patient's first degree relatives with ultrasound imaging.

## 4. Discussion

Paragangliomas of the head and neck are quite rare tumors, and although the exact incidence is unclear they are considered to represent less than 0.5% of all head and neck tumors [2]. Paragangliomas can arise from the carotid body, the tympanic, jugular, and high vagal paraganglia. The paraganglioma of the carotid body (CBP) is the most common paraganglionic neoplasm distributed in the head and neck area. Pediatric paragangliomas are even more rare entities and only a few cases have been reported in the recent literature (Table 1).

First recognized by Arias-Stella, CBPs occur in individuals under chronic stimulation by low environmental atmospheric partial pressure of oxygen, leading to hyperplasia of the chief cells of the carotid body [5]; Saldana et al. reported increased incidence of the tumor in Peruvian patients living in high altitudes and concluded that hypoxia was the primary stimuli leading to carotid body hyperplasia. This distinct disease may manifest with hyperplastic carotid body lesions under 1 cm and should be distinguished from typical CBP which should be treated surgically [6].

In our review of the literature we recognized 23 cervical paragangliomas involving the carotid body in children and adolescents. Mean patient age at the time of treatment was 12 years, with range 6 to 18 years (Table 1). There seemed to be a female predominance (female:male ratio, 2:1); however, some studies lack gender data and, thus, the ratio may be misleading.

Two of the patients had bilateral CBPs and at the same time presented jugular tumor [7, 8], while one patient was also diagnosed with a pheochromocytoma [9]. The overall incidence of multifocality in the sporadic form of the disease is estimated to be 5%, but this rises to 33% in familiar cases, where the disease is inherited in an autosomal-dominant pattern [10].

The incidence of familial paragangliomas is approximately 10%, and about 30% of them are caused by mutations in succinate dehydrogenase (SDH) gene [11, 12]. Presentation at young age could be associated with increased incidence of familial disease. In our review we identified one patient with familial disease and no patient with identified SDH gene mutations, although SDH mutation frequency in these patients may be underestimated, due to treatment during past decades. None of our patients was tested for SDH mutations, as this was not routinely performed in our unit during that period.

Diagnosis of these tumors can be quite challenging and most patients overlook the symptoms of the disease or are misdiagnosed with cervical lymphadenopathy, salivary gland pathology, neurofibromas, brachial cleft cysts, or lipomas [13]. Incorrect clinical diagnosis is reported in up to 30% of cases. Fine needle aspiration is not helpful as it is considered dangerous and is accompanied by a high risk of bleeding [1, 14]. Hogan et al. described a CBP in an 8-year-old female with a neck mass mistaken as reactive lymphadenopathy [15]. This is in accordance with the course of our second patient. In our review we found that most patients were either asymptomatic or had a palpable neck mass with recent increase in size [6, 13, 15–20].

The most important preoperative imaging is CTA or MRA, while digital subtraction angiography (DSA) is the mainstay of the diagnosis, with 100% reported accuracy, whereas the “lyre sign”, an enhancing vascular oval mass widening the angle of the bifurcation, is essentially pathognomonic of a CBP [10]. Radionuclide perfusion scanning with 99mTc pertechnetate has also been used to diagnose CBPs, with some success, while the use of meta-iodobenzylguanidine (MIBG) scanning and xenon CT have been reported [1]. In young patients imaging modalities that incorporate radiation should be minimized if possible.

Benign tumors were found in 14 patients [7, 9, 11–13, 15, 16, 18–24], while malignancy was reported in 3 patients [8, 14, 25]. One patient had a family history of disease [8]. Biological behavior of the tumor was not reported in 5 patients [4, 6, 14, 17, 26]. One patient with malignancy died within 4 months. All patients with malignant CBPs were subjected to chemotherapy or/and radiotherapy [8, 14, 25]. Most authors agree that the only proof of malignancy is the presence of metastases. Histological appearance of a CBP is not a reliable indicator of malignancy. Many clinical, histological, and molecular characteristics have been studied in order to diagnose malignant behavior, with limited clinical utility [27]. Young age, multifocality, and family history of disease seem to be associated with malignancy [1].

Complete surgical removal of the tumor is the gold standard operation. Although this is quite straightforward in Shamblin I tumors, locally advanced tumors constitute a challenge. Radical resection is crucial due to the possibility of malignancy. According to Luna-Ortiz et al., all tumors Shamblin class II and even tumors infiltrating vessels (Shamblin class III) must be managed surgically [28]. Surgical removal of CBPs has evolved from having unacceptably high rates of mortality and morbidity, to a relatively safe operation. The incidence of cerebrovascular complications has decreased from 23% to < 5% and the perioperative mortality has decreased from 6% to 0%. Desmond et al. support that meticulous hemostasis and subadventitial dissection is the key to a successful surgical intervention [29]. However, dysfunction or permanent cranial nerve impairment remains high according to recent studies [6–8, 12, 16]. Six of the young patients identified had a cranial nerve related injury [6–8, 22–24]. One of them experienced a permanent paralysis of the hypoglossal (XII) nerve [22], and multiple nerve injuries were caused in another including paralysis of the glossopharyngeal (IX), vagal (X), spinal accessory (XI), and hypoglossal (XII) nerves [7, 8]. In three cases the number or type of cranial nerve injury was not reported [6, 23, 24].

Most patients identified by our review had CBPs that were classified as Shamblin II and III [7, 8, 13, 16]. Only one has a Shamblin I tumor [7]. Preoperative embolization (PAE) was performed in 4 patients [15, 17, 20, 23], although this modality still remains controversial with varying results. The benefits of PAE focus on the reduction of operative time and blood loss. On the other hand, it does not prevent cranial nerve injury or inpatient hospital stay. On the contrary, some authors advocate that PAE may increase local complications during surgery due to the accompanying inflammatory process, which can damage the arterial wall nearby vessels [9, 16, 23, 25]. Other authors suggest that PAE could have a potential role in Shamblin III lesions and tumors involving the carotid arteries [30]. Alternatively, a proximal covered stent can be placed in the external carotid artery preventing intracranial emboli from coils during PAE [31]. In the same session, balloon occlusion of the internal carotid artery can be performed to determine if the patient can tolerate blockage of the vessel in case of ligation, clamping, or shunting [23]. The final solution in Shamblin III CBPs is removal with excision of the entire carotid bifurcation with interposition of a vascular conduit with saphenous vein or PTFE for the ICA and ligation of the ECA [30].

## 5. Conclusion

Neck paraganglioma in childhood is extremely rare incidence, with only a few published cases in literature. However, CBPs must be included in the differential diagnosis of pulsatile cervical mass and imaging with US and CT or MRI needs to follow, for evaluating the nature of the mass and the relation with the adjacent vessels and the anatomic structures.

## Figures and Tables

**Table 1:** Reported cases of CBPs in pediatric age group [4, 6–9, 11–26].

Year/author	Age/ Gender	Symptoms	Shamblin type	Size (mm)	Malignancy	Cranial nerve injury	Treatment	Follow-up period without complications (months)
1968/ Chambers et al [11]	12/M	NR	NR	25X25	No	No	Surgical Excision	120
	14/M		NR	60X60				120
	9/F		NR	15X20				12

1971/ Shamblin et al [12]	12/NR	NR	NR	NR	No	NR	Surgical Excision	NR

1980/ Newland et al [14]	13/F	Hypertension, Tachycardia	NR	25X80	NR	NR	Surgical Excision/ Radiotherapy, Chemotherapy	NR

1983/ Carney et al [21]	12/F	NR	NR	NR	No	No	Surgical Excision	Epithelioid myosarcoma- chondroma (24y), two nodules in left lung (30 y), well at age 31

1986/ Dickinson et al [22]	12/F	NR	NR	NR	No	XII permanent paralysis	Surgical Excision	NR

1988/ Hallett et al [6]	12/NR	Neck Mass Tumor	NR	NR	NR	Yes (not specified)	Surgical Excision	NR

1989/ Thompson et al [7]	14/F	Bilateral CBPs and Glomus Jugular Tumor	I	20X20	No	Right facial nerve paralysis	Surgical Excision	NR
			III	Large		No		

1990/ Gounot et al [16]	14/M	Right neck mass	II	35X25X15	No	No	Subadventitial Excision	12

1991/ Ophir et al [8]	12/F	Bilateral CBPS and Unilateral Jugular Tumor	III	30X40	Family History (+)	Paralysis of IXth to XIIth cranial nerves	Surgical Excision	96
			II	30X30		No		

1993/ Varudkar et al [9]	6/M	Swelling, Hypertension, Pheochromocytoma	NR	NR	No	No	Surgical Excision	NR

1999/ Hajnzic et al [25]	9/M	NR	NR	50	Metastatic Chest Paraganglioma, Pulmonary Metastasis	No	Surgical Excision Chemotherapy	Death after 4 months

2000/ Wang et al [23]	10/NR	NR	NR	NR	No	Yes (not specified)	Surgical Excision, Preoperative Embolization	2.9

2001/ Plukker et al [24]	11/NR	NR	NR	NR	No	Yes (not specified)	Surgical Excision	NR

2003/ Takautz et al [4]	8.2/F	NR	NR	25	NR	No	Surgical Excision	118

2005/ Osborne et al [17]	16/F	Nonpulsatile Tumor	NR	NR	NR	NR	Preoperative Emboli, Subadventitial Dissection	NR

2007/ Zaupa et al [18]	15/M	Asymptomatic Mass	NR	NR	No	NR	Subadventitial Excision	NR

2007/ Sajid [26]	18/ NR	NR	NR	NR	NR	NR	Surgical Excision	NR

2008/ Georgiadis et al [13]	13/F	Painless Pulsatile Slow-Growing Mass	III	50X43X30	No	No	En Bloc Surgical Excision	9

2014/ Lopez-vasquez et al [19]	16/F	Asymptomatic Mass	NR	40X30X30	No	NR	Surgical Excision	NR

2017/Ifeoluwa et al [20]	8/F	Pulsatile Slow-Growing Mass	NR	NR	No	No	Preoperative embolization Surgical resection	Current time

2018/Hogan et al [15]	8/F	Neck mass	NR	NR	No	No	Preoperative embolization and balloon test occlusion Surgical excision	NR

2018/ our cases	16/ F	Cervical Pain, Dizziness, Dysphagia, Breathlessness and Palpable Mass	II	26x21x30	No	No	Surgical Excision	26
	15/M	Asymptomatic Mass	II	60X35	No	No	Surgical Excision	96

M: male, F: female, and NR: not reported.

## References

[1] Dimakakos P. B., Kotsis T. E. (2001). Carotid body paraganglioma: Review and surgical management. *European Journal of Plastic Surgery*.

[2] Offergeld C., Brase C., Yaremchuk S. (2012). Head and neck paragangliomas: clinical and molecular genetic classification. *Clinics*.

[3] Woolen S., Gemmete J. J. (2016). Paragangliomas of the head and neck. *Neuroimaging Clinics of North America*.

[4] Tekautz T. M., Pratt C. B., Jenkins J. J., Spunt S. L. (2003). Pediatric extraadrenal paraganglioma. *Journal of Pediatric Surgery*.

[5] Arias-Stella J., Bustos F. (1976). Chronic hypoxia and chemodectomas in bovines at high altitudes. *Archives of Pathology & Laboratory Medicine*.

[6] Hallett Jr. J. W., Nora J. D., Hollier L. H., Cherry K. J., Pairolero P. C. (1988). Trends in neurovascular complications of surgical management for carotid body and cervical paragangliomas: a fifty-year experience with 153 tumors. *Journal of Vascular Surgery*.

[7] Thompson J. W., Cohen S. R. (1989). Management of bilateral carotid body tumors and a glomus jugulare tumor in a child. *International Journal of Pediatric Otorhinolaryngology*.

[8] Ophir D. (1991). Familial multicentric paragangliomas in a child. *The Journal of Laryngology & Otology*.

[9] Varudkar A. S., Kokandkar H. R., Gumaste G. G., Bhople K. S., Kumbhakarna N. R. (1993). Carotid body paraganglioma with coexistent pheochromocytoma in childhood. *Indian Journal of Cancer*.

[10] Davila V. J., Chang J. M., Stone W. M. (2016). Current surgical management of carotid body tumors. *Journal of Vascular Surgery*.

[11] Chambers R. G., Mahoney W. D. (1968). Carotid body tumors. *The American Journal of Surgery*.

[12] Shamblin W. R., ReMine W. H., Sheps S. G., Harrison E. G. (1971). Carotid body tumor (chemodectoma). Clinicopathologic analysis of ninety cases. *The American Journal of Surgery*.

[13] Georgiadis G. S., Lazarides M. K., Tsalkidis A., Argyropoulou P., Giatromanolaki A. (2008). Carotid body tumor in a 13-year-old child: case report and review of the literature. *Journal of Vascular Surgery*.

[14] Newland M. C., Hurlbert B. J. (1980). Chemodectoma diagnosed by hypertension and tachycardia during anesthesia. *Anesthesia & Analgesia*.

[15] Hogan A. R., Sola J. E., Jernigan S. C., Peterson E. C., Younis R. T. (2018). A pediatric carotid body tumor. *Journal of Pediatric Surgery*.

[16] Gounot E., Couillault G., Maingueneau C., Autissier J. M. (1990). Paraganglioma of the carotid body. Apropos of a case in a 14-year-old child. *Chirurgie Pédiatrique*.

[17] Osborne R. F. (2005). Nonpulsatile carotid body tumor in a teenager. *Ear, Nose & Throat Journal*.

[18] Zaupa P., Höllwarth M. E. (2007). Carotid body paraganglioma: rare tumor in a 15-year-old adolescent boy. *Journal of Pediatric Surgery*.

[19] Lopez-Vazquez M. E., Llamas-Macias F. J., Nuno-Escobar C., Gonzalez-Ojeda A., Fuentes-Orozco C., Macias-Amezcua M. D. (2014). Carotid body paraganglioma in a teenager. Case report. *Cirugia y Cirujanos*.

[20] Ifeoluwa A., Lázár I., Szövördi É., Karosi T. (2017). Management of carotid body tumor in pediatric patients: a case report and review of the literature. *International Journal of Pediatric Otorhinolaryngology*.

[21] Aidan Carney J. (1983). The triad of gastric epithelioid leiomyosarcoma, pulmonary chondroma, and functioning extra-adrenal paraganglioma: A five-year review. *Medicine (United States)*.

[22] Dickinson P. H., Griffin S. M., Guy A. J., McNeill I. F. (1986). Carotid body tumour: 30 years experience. *British Journal of Surgery*.

[23] Wang S. J., Wang M. B., Barauskas T. M., Calcaterra T. C. (2000). Surgical management of carotid body tumors. *Otolaryngology—Head and Neck Surgery*.

[24] Plukker J. T. M., Brongers E. P., Vermey A., Krikke A., Van Den Dungen J. J. A. M. (2001). Outcome of surgical treatment for carotid body paraganglioma. *British Journal of Surgery*.

[25] Hajnžić T. F., Krušlin B., Belicza M. (1999). Carotid body paraganglioma in a nine-year-old boy with extensive pulmonary metastases. *Medical and Pediatric Oncology*.

[26] Sajid M. S., Hamilton G., Baker D. M. (2007). A multicenter review of carotid body tumour management. *European Journal of Vascular and Endovascular Surgery*.

[27] Nastos C., Yiallourou A., Kotsis T. (2018). Immunohistochemical expression patterns of S100, synaptophysin, chromogranin A and neuron specific enolase in predicting malignant behaviour in paragangliomas. *Journal of B.U.ON*.

[28] Luna-Ortiz K., Rascon-Ortiz M., Villavicencio-Valencia V., Granados-Garcia M., Herrera-Gomez A. (2005). Carotid body tumors: review of a 20-year experience. *Oral Oncology*.

[29] Desmond T. H. W., Christopher H. G. (2010). Current concepts in the management of carotid body tumours. *The Medical Journal of Malaysia*.

[30] Kakkos S. K., Reddy D. J., Shepard A. D., Lin J. C., Nypaver T. J., Weaver M. R. (2009). Contemporary presentation and evolution of management of neck paragangliomas. *Journal of Vascular Surgery*.

[31] Scanlon J. M., Lustgarten J. J., Karr S. B., Cahan J. I. (2008). Successful devascularization of carotid body tumors by covered stent placement in the external carotid artery. *Journal of Vascular Surgery*.

